# Clonal Spread of *Streptococcus pyogenes* *emm*44 among Homeless Persons, Rennes, France

**DOI:** 10.3201/eid1702.101022

**Published:** 2011-02

**Authors:** Anne Cady, Céline Plainvert, Pierre-Yves Donnio, Pascaline Loury, Didier Huguenet, Alain Briand, Matthieu Revest, Samer Kayal, Anne Bouvet

**Affiliations:** Author affiliations: Centre Hospitalier Universitaire Pontchaillou, Rennes, France (A. Cady, P.-Y. Donnio, M. Revest, S. Kayal);; University Paris Descartes, Paris, France (C. Plainvert, A. Bouvet);; Université de Rennes1, Rennes (P.-Y. Donnio, S. Kayal);; Cellule de l’Institut National de Veille Sanitaire en Région Ouest, Rennes (P. Loury, A. Briand);; de l'Agence Régionale de Santé de Bretagne, Rennes (D. Huguenet)

**Keywords:** Bacteria, France, Streptococcus pyogenes, emm typing, drug users, homeless persons, skin infections, letter

**To the Editor:** *Streptococcus pyogenes,* or group A streptococci (GAS), are human pathogens responsible for pharyngitis as well as skin and soft tissue infections. Invasive GAS diseases, including bacteremia, cellulitis, and necrotizing fasciitis, are life-threatening, especially when associated with toxic shock syndrome. Several risk factors for GAS infections are known, such as diabetes, immunosuppression, drug use, and skin lesions (*1**,**2*).

In France in 2008, 12% of GAS strains were reported resistant to tetracycline by the national reference center. Unexpected recognition of 8 tetracycline-resistant GAS isolates in January and February 2009 at the 1,950-bed University Hospital of Rennes in western France led to further investigation. We report results of characterization of tetracycline-resistant GAS isolates collected during 2009 from hospitalized and outclinic patients.

Isolates were identified as GAS on the basis of β-hemolysis, Gram staining, negative catalase test result, positive pyrrolidonyl arylamidase test result, and agglutination with Lancefield group A antiserum. Antimicrobial drug susceptibility to penicillin G, amoxicillin, erythromycin, lincomycin, tetracycline, rifampin, streptomycin, kanamycin, gentamicin, and vancomycin was tested by using the disk diffusion method according to the criteria of the French Society for Microbiology (www.sfm.asso.fr). Of 72 nonduplicate GAS isolates collected, 25 (17 from inpatients, 8 from outpatients) were identified as tetracycline resistant; they were further characterized as described (*3*).

The *emm* types of these 25 tetracycline-resistant strains were determined by sequencing the variable 5′ end of the *emm* gene and comparing sequences with the Centers for Disease Control and Prevention database (www.cdc.gov/ncidod/biotech/strep/doc.htm). Twenty-three strains were *emm*44 type, 1 was *emm*105, and 1 *emm*83. Pulsed-field gel electrophoresis (PFGE) patterns obtained after DNA digestion by *Sma*I restriction enzyme were compared according to Tenover criteria (*4*). The epidemic clone including 22 strains was characterized by an identical PFGE pattern 44-A1, whereas PFGE pattern 44-A5 of the remaining *emm*44 strain differed by 4 DNA bands (Figure). Epidemic strains also shared the same biotype 3 obtained on rapid ID 32 Strep strips (bioMérieux, Marcy l’Etoile, France). T types were determined on trypsinated bacteria by slide agglutination with type-specific antisera. Eleven strains were type T11, 4 type T11/12, 1 type T11/3/13/B3264, and 6 non–T-typeable.

**Figure:**
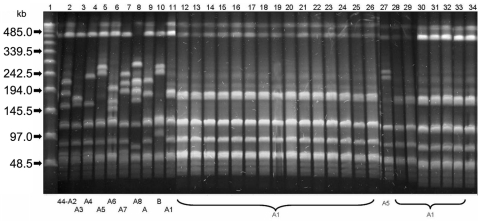
Pulsed-field gel electrophoresis (PFGE) patterns of *Sma*I-restricted chromosomal DNA of *Streptococcus pyogenes* *emm*44 strains. Lane 1, Bacteriophage Lambda ladder PFGE Marker (New England Biolabs Inc., Beverly, MA, USA); lanes 2–11, PFGE patterns 44-A2, 44-A3, 44-A4, 44-A5, 44-A6, 44-A7, 44-A8, 44-A, 44-B, and 44-A1 of *emm*44 unrelated control strains; lanes 12–26 and 28–34, 22 identical 44-A1 PFGE patterns shared by the tetracycline-resistant outbreak isolates; lane 27, PFGE pattern 44-A5 of the nonclonal *emm*44 strain isolated during the same outbreak, which differs by 4 bands from the pattern 44-A1.

All epidemic *emm*44 strains were susceptible to all antibacterial agents tested except tetracycline. MICs of tetracycline, determined with Etest method (AB Biodisk, Solna, Sweden), were 24–48 mg/L. Screening of strains by PCR for *tet*(*M*), *tet*(*O*), *tet*(*K*), and *tet*(*L*) genes showed tetracycline resistance was related to *tet*(*M*) gene. A multiplex PCR for detection of *speA*, *speB*, *speC*, *smeZ*, and *ssa* toxin genes showed that epidemic strain possessed only *speB* gene.

Investigation conducted by local health authorities showed that the first 5 patients with *emm*44 strain were drug users sharing a squat (illegally occupied housing). Although this place was shut down at the end of February after an outbreak of scabies, additional cases of infections caused by *emm*44 strain occurred. Medical care is difficult to implement for homeless persons, thus, we limited our action to swabbing symptomatic persons to treat them and to limit spread of the epidemic strain. Following recommendations from the Institute for Public Health Surveillance, in mid-April nurses at the 2 main social centers for homeless persons obtained samples from 17 persons. Eleven persons were infected with GAS, of whom 8 had not been swabbed before. All but 1 isolate was *emm*44.

Among the 22 patients infected with epidemic 44-A1 clone, 4 had several successive isolations of this strain. Most (19) infections were secondary infections of skin injuries; others were abscesses (*4*), septic arthritis (*2*), necrotizing fasciitis (*1*), erysipelas (*1*), and hygroma (*1*). Five isolates were from sterile sites (1 surgical sample of necrotizing fasciitis, 1 blood culture, and 3 joint fluids). Most infections had favorable outcomes, with the exception of a 79-year old man who died of erysipelas. Patient median age was 37 years (range 20–79 years); all but 1 were men. Eighty-six percent had risk factors such as alcohol abuse (17, 77%), homelessness (16, 73%), drug use (11, 50%), hepatitis C infection (4, 8%), and HIV infection (1, 4.5%). Two patients had no identified risk factors. Complete characteristics of 50 patients infected with a strain of GAS different from 44-A1 clone were not available. However, this population did differ by its sex ratio (28 men:22 women) and by older median age (47.^3^ years).

We report clonal spread of an *emm*44 tetracycline-resistant GAS strain in marginal populations (drug users and homeless persons) in Rennes. This strain, characterized by PFGE pattern 44-A1, represented 22/25 tetracycline resistant GAS isolates and 30% of the 72 GAS isolates identified at the hospital in Pontchaillou in 2009. Locally, emergence of the 44-A1 clone led to the dramatic increase of GAS tetracycline resistance, from 17% in 2008 to 35% in 2009. *emm*44 GAS strains, which share identical 5′*emm* sequences with previously designated M/ *emm*61 strains (*5*), have mainly been isolated in Asia from throat and skin specimens (*6**,**7*). They were rarely reported as responsible for invasive infections in France or other parts of the world (*5**,**8*). Polyclonal and *emm*25 and *emm*83 monoclonal GAS outbreaks have been recently described among drug users in Switzerland, the United Kingdom, and Spain (*9**,**10*) without robust evidence of enhanced virulence of the causative GAS strains. In the outbreak we report, skin infections might be a leading cause of bacterial transmission between people living in poor hygienic conditions and overcrowded spaces.
